# Comment on “Human Cardiovascular Organoids: Biomedical Applications and Ethical Challenges”

**DOI:** 10.1016/j.ahjo.2026.100983

**Published:** 2026-10-21

**Authors:** N. Muninathan, V. Ramnath, Mohana Thiruchenduran, M. Kavitha

**Affiliations:** a Central Research Laboratory, Meenakshi Medical College Hospital & Research Institute, Meenakshi Academy of Higher Education and Research, Enathur, Kanchipuram, Tamil Nadu, 631552, India; b Meenakshi College of Allied Health Sciences, Meenakshi Medical College Hospital & Research Institute, Meenakshi Academy of Higher Education and Research, Enathur, Kanchipuram, Tamil Nadu, India; c Department of Biochemistry, Meenakshi Ammal Dental College and Hospital, Meenakshi Academy of Higher Education and Research, Chennai, Tamil Nadu, India; d Meenakshi College of Nursing, Meenakshi Academy of Higher Education and Research, Enathur, Kanchipuram, Tamil Nadu, India

**Keywords:** Cardiovascular organoids, Cardiac organoids, Engineered heart tissue, iPSC, Heart-on-chip, Biomedical applications, Disease modeling, Gene therapy, Ethical challenges, Organoid research

Dear Editor,

Sen et al. [1] start with a helpful correction. They say that different types of platforms are often grouped together as organoids even though they are different in where they come from and how much control is used in making them. They also say that this grouping causes people to think these models are more similar to hearts than they really are. They then offer a way to classify them: self-organizing organoids come from the natural development process while engineered heart tissues, bioprinted models and organ-on-chip systems have their shape made from the outside. They say these should be called engineered myocardium of organoids.

The rest of the review does not follow that classification [2]. The title, the abstract and the sections about applications all talk about organoids while using evidence from the systems that the classification excludes. The part about arrhythmia models uses microelectrode arrays and biomechanical engineered tissues. The work on hypertrophy comes from engineered tissues that are exposed to adrenergic signals. The data on Barth syndrome gene therapy comes from a heart-on-chip platform. The section about myocardial repair talks about cardiac muscle patches. Each of these is described correctly where it is. Then it is put into statements about what organoids can do. The ethics section does the same talking about rules for organoid material without making a difference between a self-organizing heart structure and a collagen scaffold with heart cells. A person who takes the classification seriously would see the review making the mistake it warned about [3]. Using the platform label in every claim about what can be done would fix this. Would make the paper much more helpful than most in this busy field.

The ethical part needs a kind of focus. Most of what's there could apply to any iPSC work: ways to get consent, privacy of genetic information storing samples selling them making sure people can access them. What is unique to this area is not looked at closely. Self-organizing heart organoids make chambers. Beat by themselves and some groups are now making models that have more than one type of cell including heart, blood and nerve cells [4]. Whether these models bring up issues beyond tissue rules is still being discussed and the reviews own description of models that make blood and form hearts and models with nerve cells brings this up. The problem of cells that can cause heart issues, which the paper mentions as a challenge for using these models is also a safety issue for patients. This should be in the ethics section. Not just listed as a technical problem.

One statement in the part should be taken back. The authors say that the main research they talk about was done under systems that ask for consent respect for the donor and keep people involved through dynamic consent. Across about two hundred references this can't have been checked. Dynamic consent is still not common in practice. The review itself says that such models have only been suggested. Saying that consent rules vary across the research they mention would be correct. Would not cost anything.

Lastly the part about injury in the disease modeling section says the same thing twice in a row. The first time it is attributed to WNT-modulated cardioids and the time, to oxygen-gradient platforms. This part needs to be separated so each result is connected to the system that made it.

## CRediT authorship contribution statement

**N. Muninathan:** Conceptualization. **V. Ramnath:** Data curation. **Mohana Thiruchenduran:** Writing – review & editing. **M. Kavitha:** Writing – original draft.

## Ethical approval

Not applicable (commentary on previously published research).

## Declaration of Generative AI and AI-assisted technologies in the writing process

Generative AI tools were used solely for language refinement and formatting assistance. All scientific interpretation and critique were independently developed by the authors.

## Funding

None.

## Declaration of competing interest

The authors declare no conflicts of interest.

## Data Availability

No new data were generated or analysed in this work.
